# Cross-reactivity of outer membrane proteins of *Campylobacter* species with cholera toxin

**Published:** 2011-02

**Authors:** M. John Albert

**Affiliations:** *Department of Microbiology, Faculty of Medicine, Kuwait University, Jabriya, Kuwait*

**Keywords:** campylobacter, cholera toxin, like toxin, GM1 ELISA, PorA

## Abstract

*Campylobacter jejuni* is a foodborne pathogen and a leading cause of diarrhoea worldwide. It is believed that a cholera toxin-like toxin (CTLT) produced by *C. jejuni* may mediate watery diarrhoea. However, the production of a CTLT by *C. jejuni* is controversial. A cholera toxin gene (*ctx*) homologue has not been identified in *Campylobacter* species. We investigated the identity of the CT cross-reactive antigen from *Campylobacter* species previously and the results are reviewed here. Filtrates of *C. jejuni* grown in four different liquid media, reported to promote CTLT production, were tested by Chinese hamster ovary (CHO) cell elongation assay for functional toxin and for reactivity with CT antibody using GM1 ganglioside ELISA (GM1 ELISA) and immunoblotting. Protein sequence of the CT antibody-reactive band was determined by matrix-assisted laser desorption ionization-time of flight (MALDI TOF-TOF). Non-*jejuni* species (*C. coli, C. lari, C. foetus, C. hyointestinalis and C. upsaliensis*) were investigated by CHO cell assay and immunoblotting. Filtrates from seven *C. jejuni* reference strains reported to produce CTLT and from 80 clinical strains were negative in the CHO cell assay. However, filtrates from three reference strains and 16 clinical strains were positive by GM1 ELISA. All strains irrespective of GM1 ELISA reactivity, possessed a 53-kDa protein which reacted with CT antibody by immunoblotting. This band was identified as the major outer membrane protein (PorA) of *C. jejuni*. CT antibody reacted with a *C. jejuni* recombinant PorA on immunoblotting. All non-*C. jejuni* strains were negative by CHO cell assay, but the common 53-kDa proteins reacted with CT antibody on immunoblots. The cross-reactivity of PorAs of *Campylobacter* species with CT may lead to the erroneous conclusion that *Campylobacter* species produce a functional CTLT.

## Introduction

*Campylobacter jejuni* a foodborne pathogen, is a leading cause of bacterial diarrhoea worldwide^1^. It causes mostly inflammatory diarrhoea in individuals in developed countries, but watery diarrhoea in individuals in developing countries^2^. A cholera toxin-like toxin (CTLT) produced by *C. jejuni* was thought to mediate watery diarrhea^3^. However, CTLT production by C. jejuni is controversial. While some investigators reported it, others did not^3^. A cholera toxin gene (ctx) homologue has not been found in C. jejuni^3^. There are also reports of CTLT production by non-*C. jejuni* species, especially *C. coli, C. lari* and *C. hyointestinalis*^4^,^5^. We recently reexamined the question of CTLT production by *C. jejuni* and other species and identified the antigen that cross-reacts with CT antibody^6^–^8^. This article summaries many findings on this line of research.

## Experimental details

We studied a number reference and clinical isolates. Reference *C. jejuni* cultures that had been reported to produce CTLT included the following strains: CCUG 8731, CCUG 6951, CCUG 6968 and CCUG 8680 (received from the University of Goteborg culture collection, Goteborg, Sweden); 180 ip and 189 ip (kindly provided by G. Ruiz-Palacios, National Institute of Medical Science and Nutrition, Mexico Districto Federal, Mexico); and CJ0094400 (kindly provided by A. Lee, University of New South Wales, Sydney, Australia). A *ctx* gene negative, fully sequenced *C. jejuni* strain NCTC 11168 was obtained from B. W. Wren (London School of Hygiene and Tropical Medicine, London, United Kingdom). Clinical *C. jejuni* strains included 10 strains from the International Centre for Diarrhoeal Disease Research, Bangladesh, Dhaka, Bangladesh (kindly provided by M. Rahman) and 70 strains from patients treated at Mubarak Al-Kabeer Hospital, Jabriya, Kuwait. Two strains each of *C. coli, C.lari, C. foetus, C. hyointestinalis* and *C. upsaliensis* were provided by G. Hogg (University of Melbourne, Victoria, Australia). An enterotoxigenic *Escherichia coli* (ETEC) strain, H10407, producing heat-labile toxin (LT) served as a positive control for enterotoxin production. It is well known that the NH2-terminal regions of LT and CT exhibit the highest degree of homology (91%), while the COOH-terminal region, containing the sole cystine residue in each toxin is less conserved (~52%).

All strains were screened in Casamino Acids–yeast extract (CAYE) broth supplemented with 1.0 μg/ml ferric chloride (CAYEF medium) for functional CTLT production. In addition, selected *C. jejuni* strains were screened in (*i*) Brucella broth supplemented with 0.25 per cent each of L-asparagine, L-serine and L-glutamic acid (BASG broth), (*ii*) BASG broth supplemented with 0.05 to 0.5 per cent L-cysteine, corn starch, yeast extract and dextrose, and (iii) G-C medium supplemented with 0.1 per cent IsoVitalex (GCIV medium). The effect of polymyxin B on release of CTLT from periplasmic space was studied with cultures grown in GCIV medium. Cultures were grown microaerobically under shaking or stationary conditions at 42°C for 24-48 h.

Selected *C. jejuni* strains were passaged multiple times through the intestinal loops of Sprague-Dawley rats and the secreted intestinal fluids were tested for the presence of functional toxin. Chinese hamster ovary (CHO) cell elongation assay was used for the detection of functional CTLT. Serial doubling dilutions of bacteria-free culture supernatant were used with a starting dilution of 1:2. In some cases, filtrates were concentrated to 50X by lyophilisation followed by reconstitution and dialysis before testing. Elongation of >50 per cent of the monolayer at a filtrate dilution of ≥1:4 was considered positive for CTLT production.

Serological testes used for the detection of CT crosss-reactive material in the culture filtrates were (*i*) ganglioside GM1 ELISA in which ELISA plate coated with GM1 ganglioside was reacted with culture supernatant and rabbit polyclonal CT antibody, (*ii*) direct ELISA in which ELISA plate was directly coated with bacterial sonicate and reacted with CT antibody, and (*iii*) immunoblot assay in which bacteria-free culture filtrate, crude outer membrane protein (OMP), or sarcosyl-purified OMP was separated by SDS-PAGE, transferred to nitrocellulose membrane and probed with rabbit anti-CT antibody.

The amino acid sequence of the CT cross-reactive band was analysed by matrix-assisted laser desorption ionization-time of flight (MALDI TOF-TOF). The protein identified was further confirmed by immunoblot analysis in which a recombinant *C. jejuni* PorA protein (fusion protein, GST-PorA expressed from *porA* gene of *C. jejuni* strain C31) was checked for its reactivity with rabbit CT polyclonal antibody and vice-versa in which rabbit antibody to recombinant porA was checked for its activity against CT.

## Results

None of the filtrates of bacteria grown in different media was positive for CTLT production by the CHO cell assay. Fifty times concentrated culture filtrates from a Swedish strain and the Australian strain tested in this assay were negative. The control ETEC strain used in this assay was positive. The Australian strain and a Kuwaiti strain gave a fluid to loop-length ratio of 0.3 after four rat ileal loop passages. However, the ileal loop fluids did not cause CHO cell elongation. In the GM1 ELISA, CAYE filtrates from two Swedish strains, the Australian strain and 16 Kuwaiti strains were positive^7^.

Numerous strains (both GM1 ELISA positive as well as negative) and the NCTC 11168 strain (a known CT-negative strain) yielded two prominent bands of 78.6 kDa and 53 kDa size on immunoblot with rabbit anti CT antibody. However, when blots were probed with normal rabbit serum, the 78.6 kDa band only was visible. This suggested that the 53 kDa band was specifically recognized by the anti CT-antibody. This 53 kDa specific protein was subsequently identified as the MOMP (porA) of *C. jejuni*, by MALDI TOF-TOF analysis. The identity of the protein was further confirmed when the recombinant PorA protein expressed as GST-porA from the porA gene of *C. jejuni* strain (C31) reacted with rabbit anti-CT antibody on immunoblot assay. However, a rabbit polyclonal antibody raised against the GST-PorA failed to react with-CT in the immunoblot. This suggested that the reaction was a one-way reaction^7^.

Direct ELISA was performed to check for CT cross-reactivity with sonicated extracts from all 7 reference strains from Sweden, Mexico and Australia, CT-negative strain, NCTC 11168, 5 Kuwait strains from among 16 GM1 ELISA positive strains and 5 GM1 ELISA negative Kuwaiti strains. All strains were positive in the direct ELISA for CT cross-reactivity. Since the cross-reacting material is PorA, it is present in sufficient quantity in bacterial sonicates used in the direct ELISA for all strains to be positive. However, in the GM1 ELISA where culture filtrates were used, only some strains would have shed the membrane protein to be positive in the assay^7^.

Two strains each of *C. coli, C. lari, C. foetus, C. hyointestinalis* and *C. upsaliensis* were negative for CTLT production when culture filtrates from CAYE medium were tested in the CHO cell assay. Immunoblot analysis of crude OMPs revealed a common 53 kDa band for all species and several additional bands (six for *C. foetus*). When reacted with normal rabbit serum, a common non-specific band of 79 kDa for all species with two additional higher molecular weights for *C. upsaliensis* were visible. Apart from the 79 kDa non-specific band, the other six bands for *C. foetus* appeared to be specific. When Sarkosyl-purified OMPs were allowed to react with anti-CT antibody in immunoblot assays, all species (except *C. foetus*) produced the unique single band of 53 kDa. The purified OMP preparation from *C. foetus* produced several bands in a ladder-like pattern (*C. foetus* is known to possess an S-layer OMP which separates into a ladder-like pattern on electrophoresis), as did the crude membrane preparation. However, the nonspecific band of 79 kDa that reacted with normal rabbit serum was absent. When Sarkosyl-purified OMPs were allowed to react with normal rabbit serum in immunoblot assays, no band from any species was visible^8^.

## Discussion

There are reported similarities in PorA across many *Campylobacter* species at genetic and amino acid sequence levels. Therefore, the 53 kDa protein from non-*C. jejuni* species that cross-reacted with anti CT antibody is likely to be PorA.

Evidence for the production by *C. jejuni* of an enterotoxin that is structurally and biologically related to CT and LT was based on the following observations: (*i*) *C. jejuni* causes morphological alteration and raises intracellular level of cAMP in CHO and Y-1 cells^4^,^9^; (*ii*) the effects on morphology and cAMP levels in CHO and Y-1 cells are neutralised by antiserum to CT^4^,^9^; (*iii*) *C. jejuni* induces fluid secretion in rat ileal loop assay^9^,^10^; (*iv*) children infected with *C. jejuni* produce serum antibodies that cross-react with CT and LT^11^–^13^; (*v*) culture filtrates reacted in GM1 ganglioside ELISA with antiserum to CT or LT^14^–^15^; and (*vi*) galactoside, ganglioside or CT antibody affinity purified material from culture filtrates caused morphological alteration and raised cAMP levels in sensitive cells or reacted in GM1 ganglioside ELISA^15^–^17^.

However, a careful appraisal of the literature suggests that there are problems with the assays which gave rise to these findings. For morphological assay using CHO cells, no standard criterion was set for interpreting positive results. Several studies defined elongation of any number of cells (ranging from a few to <50%) as positive for the presence of an enterotoxin^4^,^18^–^20^. In other studies, either undiluted culture filtrate or filtrate at a dilution of <1:2 was used^4^,^21^,^22^. In all these studies, the amount of toxin reported to be produced by the isolates was very small. This is contrary to *V. cholerae* and enterotoxigenic *E. coli* infections, where the organisms produce large amounts of enterotoxins that result in profuse watery diarrhoea, which is not seen in *C. jejuni* infection^2^. We believe an accepted criterion for positive test would be a culture filtrate dilution of >1:4 affecting > 50 per cent of the cells. This should rule out nonspecific effects of components in the media, since undiluted or lower dilutions of the media were shown to give false positive results^23^. By this criterion, all strains tested by us were negative. Filtrates of cultures produced in different media that were reported to promote enterotoxin production failed to yield positive results.

Another factor to be considered is the confounding effect of cytolethal distending toxin (CLDT) in the CHO cell assay. Both CT and CLDT cause elongation of cells after 24 h of incubation, but the toxins are distinguished on further incubation of cells. Whereas CLDT causes progressive distention and cytotoxicity of cells over the next four days, such effects are not seen with CT^5^. Many reports on the production of CTLT by *C. jejuni* appeared before the existence of CLDT was known^9^,^20^,^24^. It is quite likely that the effect of CLDT would have been confused with that of CTLT.

The amount of cAMP reported to be produced by the *C. jejuni* isolates in cells is small in comparison to that produced by CT or LT^4^,^9^. There is only partial abrogation or no abrogation of the effect of the toxin by anti-CT and anti-LT sera on morphological assay using cell cultures^4^,^18^. However, the effect of normal serum on neutralisation of morphological change in these studies is not known. CLDT also induces modest increase in cAMP levels^5^. However, CT and CLDT are antigenically distinct^5^. As reported by others^9^,^10^, two of our isolates induced fluid accumulation in rat ileal loop assay, but only after repeated passage. However, as observed in another study^25^, the secreted fluid did not induce elongation of CHO cells, a finding consistent with the notion that fluid secretion was not due to CTLT production. It could be due to other mechanisms such as via inflammatory mediators of secretion^26^.

The evidence put forward to support the production of enterotoxin by *C. jejuni* was the development of serum antibodies to CT in children in Mexico^13^ and Central African Republic^12^ who suffered from *C. jejuni* diarrhoea. However, other studies failed to demonstrate such antibodies^27^,^28^ or demonstrated a weak antibody response only^11^. It is possible that children showing antibody responses were concomitantly infected with enterotoxigenic *E. coli*^28^. The reported purification studies of a postulated enterotoxin from *C. jejuni* have produced confusing results^10^,^15^,^16^,^29^. These studies used ammonium sulphate precipitation, followed by purification with affinity chromatography using galactose, gangliosides or CT antibodies. The purified fractions had either a single band of ~ 70 kDa^15^,^17^ or multiple bands of 68-, 54- and 43-kDa^16^,^30^. In another study, the molecular masses of bands that reacted with anti-CT antibody were > 90 kDa and 50 kDa^29^. Some bands had biological activity^15^,^16^ while others did not^16^,^30^. It is thus difficult to reconcile the findings from different studies. In our immunoblot studies with CT antibody, two bands of 78.6 kDa and 53 kDa were visible. However, the high molecular weight band was due to a non-specific reaction with normal rabbit serum.

## Conclusions

We have screened numerous, freshly isolated clinical local strains and also control strains of *C. jejuni* from various laboratories, which reportedly produced an enterotoxin. In spite of using several media that were reported to promote enterotoxin production by *C. jejuni*, none of the strains produced any enterotoxin in our hands. Thus our findings are similar to those of several other groups^11^,^23^,^27^,^28^. Other *Campylobacter* species also did not produce a functional enterotoxin by CHO cell assay in this study. One of the reasons that contributed to the conclusion that *C. jejuni* and other species produce an enterotoxin resembling CT was the reactivity of culture filtrates with anti-CT antibody in GM1 ELISA. Our results show unequivocally that *Campylobacter* species possess an antigen that reacts with anti-CT antibody. That antigen is the major outer membrane protein, PorA.
